# Effects of concomitant use of hydrogen water and photobiomodulation on Parkinson disease: A pilot study

**DOI:** 10.1097/MD.0000000000024191

**Published:** 2021-01-29

**Authors:** Chien-Tai Hong, Chaur-Jong Hu, Hung-Yu Lin, Dean Wu

**Affiliations:** a Department of Neurology, Taipei Medical University Shuang Ho Hospital, New Taipei City; b Department of Neurology, School of Medicine, College of Medicine, Taipei Medical University; c National Taipei University of Technology, Taipei, Taiwan.

**Keywords:** hydrogen water, Parkinson disease, photobiomodulation

## Abstract

**Background:**

Parkinson disease (PD), the second most common neurodegenerative disease, has no cure or applicable disease-modifying approach, only symptomatic therapy. Oxidative stress and mitochondrial dysfunction play key roles in PD pathophysiology. Animal studies have demonstrated that photobiomodulation (PBM) may enhance mitochondrial function and boost adenosine triphosphate production, thus alleviating PD symptoms; however, this process can cause increased reactive oxygen species (ROS) production. Molecular hydrogen (H_2_) is a potent and possibly therapeutic antioxidant that can mitigate the effect of ROS. PBM targeting the brainstem may facilitate neuronal activity, and the concomitant H_2_ may clear additional ROS produced by PBM. Therefore, this study aimed to determine the safety and effectiveness of PBM + H_2_ in patients with PD.

**Methods:**

We included 18 patients with PD (age 30–80 years) who were at Hoehn and Yahr stages II-III. All the participants received daily PBM + H_2_ therapy for 2 weeks. The adverse event and the Unified Parkinson Disease Rating Scale (UPDRS) scores were recorded.

**Results:**

We noted that the UPDRS scores began significantly decreasing from the first week, and this improvement persisted until the end of therapy. Moreover, no adverse event was recorded. After 1 week of therapy cessation, UPDRS scores slightly increased but the improvement remained significant compared with the baseline.

**Conclusion:**

This novel, proof-of-concept study demonstrated that PBM+H_2_ therapy is safe and reduces disease severity. A larger-scaled clinical trial is warranted to completely investigate the effects of PBM + H_2_ therapy on PD.

## Abstract 2

Supplemental Digital Content is available in the text

## 1 Introduction

Parkinson disease (PD), the second most common neurodegenerative disease, affects approximately 1% of people aged >60 years.^[1]^ Currently, the only available modality for PD management is symptomatic and is mainly based on exogenous dopaminergic supplement. No cure or disease-modifying approach to halt PD progression is available. In most people, PD progression leads to the impairment of the quality of life and a large economic burden on the patients themselves, their families, and the whole society.^[2,3]^

Since the discovery of laser in the 1960s, laser therapy has been reported to have the potential to improve wound healing and reduce pain, inflammation, and swelling (review by Lemes et al).^[4]^ Some animal studies have determined that red to infrared light or photobiomodulation (PBM) is neuroprotective in patients with PD.^[5–7]^ Low-level PBM, involving the application of red to near-infrared light (600–1000 nm) at a power density of 1 to 5 W/cm^2^, has been clinically applied globally for many disorders that require tissue healing and regeneration and tissue death prevention.^[8]^ Although the basic mechanisms underlying the beneficial effect of PBM is unclear, the mechanisms possibly involve the improvement of mitochondrial function and cellular metabolism,^[9,10]^ which is in contrast to the main PD pathogeneses involving mitochondrial dysfunction.^[11]^ The other major PD pathogenesis mechanism is the excessive net production of reactive oxygen species (ROS) and oxidative damage. Dysfunctional mitochondria generate overwhelming levels of ROS through the electron transport chain, causing oxidative stress and damage and triggering the apoptosis of the dopaminergic neurons in the substantia nigra. Antioxidative therapy has long been expected to attenuate PD risk and progression.^[12]^ Molecular hydrogen (H_2_) has recently been found to be a potent and possibly therapeutic antioxidant in both in vitro and in vivo studies (review by Ohta).^[13]^ H_2_ application could be easily achieved by drinking H_2_-dissolved water (H_2_ water). A randomized, double-blind study determined that drinking 1000 mL**/**d of H_2_-water for 48 weeks reduced PD severity.^[14]^

Despite the abundant available knowledge on PD, no single treatment aiding disease modification is available. A possible reason for this failure is the multifactorial characteristics of PD.^[15]^ Numerous pathogeneses are involved in PD development and progression, and thus a single-targeted treatment in a clinical trial cannot alleviate the accumulative damage from various sources. In theory, a simultaneous approach that targets multiple sources could be used to resolve the aforementioned concerns, but unexpected adverse effects due to the interaction of combined treatments may occur during clinical trials. Noninvasive or minimally invasive approaches are preferable for a combined therapy because of the well-tolerated safety profile.

Therefore, the present study aimed to test the safety and effectiveness of the combination of PBM and H_2_ (PBM + H_2_) for noninvasively improving mitochondria function and suppressing oxidative stress in PD. The present study was initiated by one of the authors (HYL) who has had PD for a long time. The author was able to considerably mitigate PD symptoms through the use of PBM + H_2_. (Details in Supplemental Video Clip 1 to 4, http://links.lww.com/MD/F560, http://links.lww.com/MD/F561, http://links.lww.com/MD/F562, http://links.lww.com/MD/F563 and video clip description, http://links.lww.com/MD/F564) Therefore, this study aimed to assess whether the synergistic effect can be clinically observed through the combined treatment approach.

## 2 Methods

### 2.1 Study design

**Figure 1:**
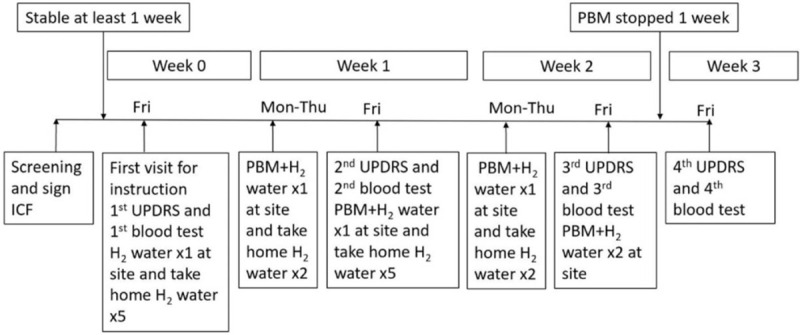
Scheme of the study design. Participants fulfill the criteria of inclusion and exclusion, the ICF of this study was explained and signed. After a week of stable condition (without any change of anti-PD medications or other active medical conditions), the participant will visit our study site for detail instructions of the study on Friday of Week 0. The first UPDRS was scored and blood was drawn for laboratory study. Hydrogen water was supplied, including those to be used during weekend at home. On Week 1 and Week 2, through Monday to Friday, the participant received daily PBM and hydrogen water at study site under the help of our study assistant. On each Friday of Week 1 and 2, UPDRS was recorded and blood sample was collected. On Week 3, the PBM and hydrogen water consumption was ceased. Another UPDRS score and blood sample was collected on Friday before the end of the study. ICF = informed consent form, PBM = photobiomodulation, UPDRS = Unified Parkinson Disease Rating Scale.

This was a small-scale, open-label, single-arm, phase-I/IIa study. All the participants received daily H_2_ water and PBM simultaneously for 2 weeks. The adverse events and the Unified Parkinson Disease Rating Scale (UPDRS) scores were recorded. Because the purpose of Part IV of the UPDRS is detecting the complications of conventional anti-PD medication use, it was omitted here. The detailed timing of PBM administration and H_2_ water consumption is illustrated in Figure 1. This study was approved by the Ministry of Health and Welfare of Taiwan (Case No.: 1070003422) and the JIRB of Taipei Medical University (Case No.: N201803065).

### 2.2 Patient and public involvement

The inclusion criteria of the study were as following:

(1) PD patients at Hoehn and Yahr (H&Y) stage II and III by a board-certified neurologist.
(2) Age between 30 and 80
(3) Generally healthy as indicated by recent physical examination
(4) If taking any psychotropic medications should be stable in the past 2 months.

In total, 18 people aged 50 to 78 years old (diagnosed as having PD at H&Y stage II-III by board-certified neurologists on the basis of the UK PD Society Brain Bank Diagnostic Criteria were recruited (Table 1). We intentionally exclude PD patients at H&Y stage I, IV, and V because we considered that stage I is too mild and stage IV and V are too severe to show possible effectiveness of our therapy. After reviewing by CT-H and D-W, all of them met the MDS-PD criteria for the diagnosis of PD as well. Only one of the participants fulfil the criteria declined to participate because objection of his family. Except for PD, these people were healthy. If they were taking any psychotropic medications, their dosage over the past 2 months had been stable. The development of the research question and outcome measures were informed to the study participants through the oral explanation and the written informed consent. The study participants were recruited from the outpatient clinic in Shuang Ho hospital and were not involved in the recruitment to and conduct of the study. The results of study were disseminated to the study participants by the principal investigator, Dr Wu in the outpatient clinic.

**Table 1:** Demographic data of the study participants (n = 17).

	Mean ± SD or number (%)
Age (yr-old)	67.53 ± 8.83
Female	6 (35.3)
Disease duration (yr)	6.15 ± 3.71
Hoen and Yahr stage	
II	11 (64.7)
III	6 (35.3)

SD = standard deviation.

During the 2 weeks before and after and the whole study period, the anti-Parkinsonism medication and antioxidant supplements were unchanged. Patients with multiple infarction, Alzheimer disease, uncontrolled or unstable chronic illness, actively growing intracranial pathology, associated psychotic illness, or systemic malignancies were excluded. All participants provided written inform consent.

### 2.3 PBM

**Figure 2:**
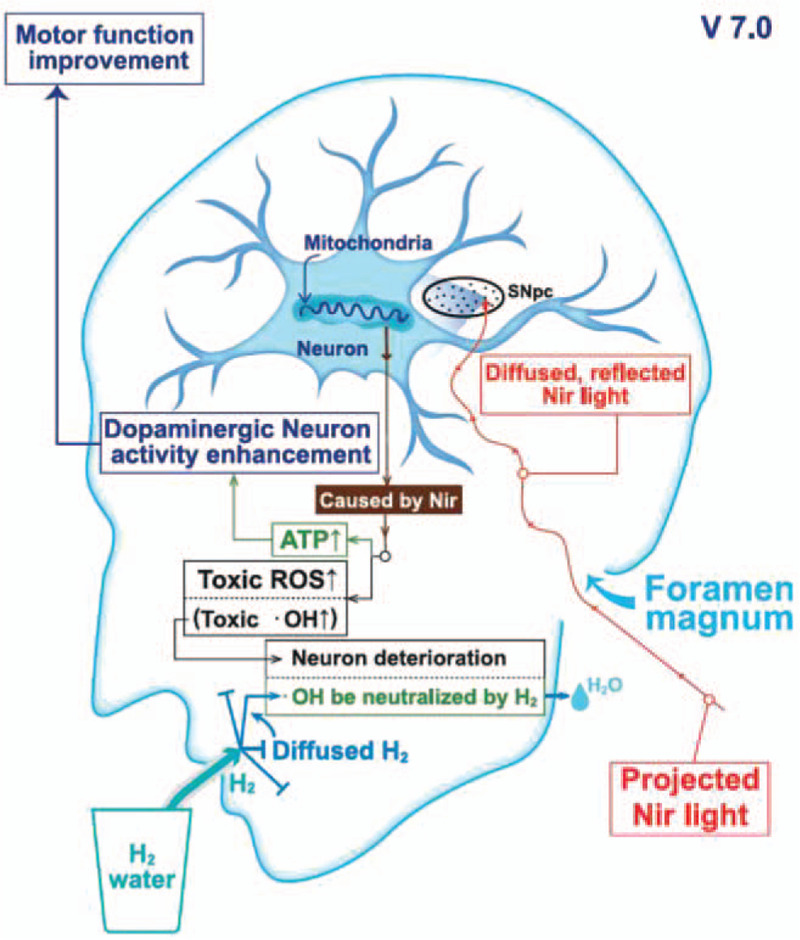
Proposed mechanism of PBM and hydrogen water. PBM = photobiomodulation.

The PBM apparatus was self-designed and manufactured by the original idea initiator (HYL). The light is composed of a light-emitting diode array (Model-102 NIR) with near-infrared (940 ± 10 nm) wavelength and intensity of 6.0 mw/cm^2^ ± 10% with a 56.7-mA current. The light is designed to be placed on the posterior aspect of the neck midline, pointing to the midbrain (illustrated as Fig. 2; Supplemental Figure shows PBM on one of the participants during treatment, http://links.lww.com/MD/F565). Light therapy was conducted for 5 consecutive days (Monday–Friday) for each patient under the monitoring and assistance of a study nurse for 2 consecutive weeks. The light source was a light-emitting diode array with no direct skin contact. The estimated temperature elevation was approximately 1°C to 3°C after a 30-minute exposure.

### 2.4 H_2_ water

The H_2_ water, I-MIZU PLATINUM, was purchased from I-MIZUCARE and imported legally from Japan with the sole purpose of being used in this study. This bottled H_2_ water is commercialized in the Japanese market under their domestic regulations. Each can of H_2_ water is 200 mL in volume and contains 2.5 ppm of dissolved hydrogen in concentration.

### 2.5 Primary endpoint and statistical methods

The primary endpoint of this study was the improvement of the sum of Parts I, II, and III of the UPDRS and its subscales (Parts I, II, and III). The demographic and basic characteristics are presented as means ± standard deviations and numbers (percentages) for continuous and categorical variables, respectively. The Kolmogorov–Smirnov test was initially used to examine the normality of data. One-way analysis of variance (ANOVA) was performed to determine the differences in demographic characteristics and relevant parameters, such as UPDRS, across various groups. For normally distributed parameters, a mixed-design ANOVA (repeated measure) with the factor time (pretreatment, week-1 and -2 treatment, and posttreatment) as a within-subjects factor and factor Group (H_2_ water and PBM) as a between-subjects factor was used to determine differences in measured data among the groups over time. For nonnormally distributed parameters, log transformation was used to normalize data for parametric analyses. Post hoc comparisons with Bonferroni correction were used to identify when differences occurred.

All statistical analyses were performed using SAS (version 9.3; SAS Institute Inc., Cary, NC), and a *P* of <.05 was considered statistically significant.

## 3 Results

**Figure 3:**
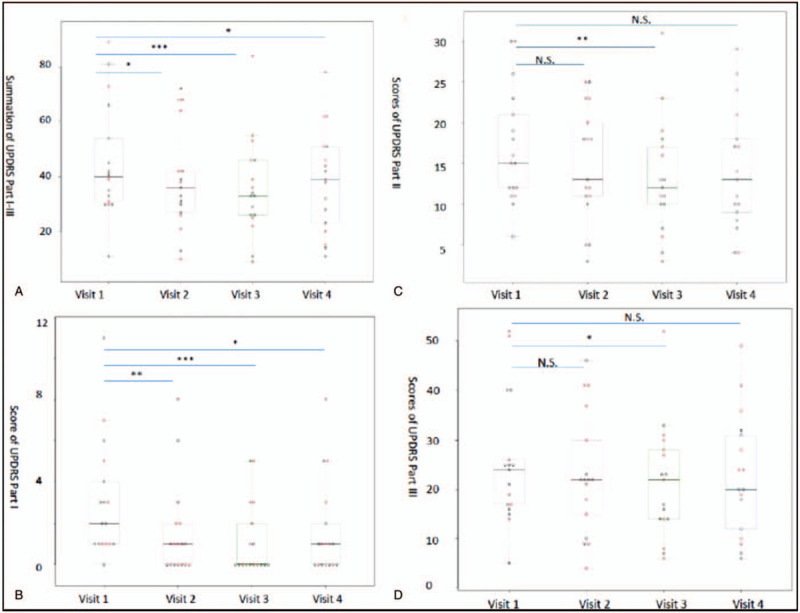
The alteration of the Unified Parkinson Disease Rating Scale (UPDRS) scores in the summation of Part I, II, III (A), Part I (B), Part II (C) and Part III (D). ^∗^*P* < .05; ^∗∗^*P* < .01; ^∗∗∗^*P* < .001.

The overall PD severity (sum of UPDRS Part I, II, and III scores) significantly decreased after only 1 week of PBM + H_2_ (Fig. 3) (detail in Supplementary Table 1, http://links.lww.com/MD/F566). This improvement continued over the second week of therapy. After cessation of therapy for 1 week, the total score increased but still exhibited significant improvement compared with the initial score. The Part I scores significantly decreased after only 1 week of PBM + H_2_. This improvement continued after the second week of therapy. After the cessation of therapy for 1 week, Part I scores increased but still demonstrated significant improvement compared with the initial scores. For Parts II and III, the scores slightly but not significantly decreased after 1 week of PBM + H_2_. However, the scores decreased considerably after 2 weeks of PBM + H_2_. After the cessation of therapy for 1 week, the score remained slightly decreased compared with the initial score but not significantly. For each of the subitems, we did one-way ANOVA post-hoc analysis and it shows significant improvement of the intellectual impairment and depression of Part I, falling (unrelated to freezing) of Part II and tremor of Part III as compared to the baseline (Supplementary Table 2, http://links.lww.com/MD/F567).

During the course of PBM and hydrogen water therapy of our study, there were no adverse event observed or reported by the participants or team members. The results of blood test before and after the PBM + H2 water treatment are shown in Table 2. There are no remarkable changes before and after treatment observed.

**Table 2:** The blood tests data of study participants.

	Baseline	End of 1st wk PBM + H_2_ water	End of 2nd wk PBM + H_2_ water	Cessation of therapy for 1 wk
BUN (mg/dL)	15.71 ± 3.58	14.94 ± 3.88	14.24 ± 3.35	14.18 ± 3.20
Creatinine (mg/dL)	0.92 ± 0.23	0.92 ± 0.23	0.92 ± 0.25	0.91 ± 0.22
GOT (U/L)	24.35 ± 4.03	22.59 ± 4.03	23.71 ± 6.04	23.65 ± 5.28
GPT (U/L)	20.47 ± 8.32	19.41 ± 9.37	18.12 ± 7.71	18.76 ± 8.44
WBC (10^3^/uL)	5.85 ± 1.01	6.12 ± 0.91	5.92 ± 1.05	6.04 ± 1.17
Hb (g/dL)	13.56 ± 1.71	13.89 ± 1.58	13.66 ± 1.58	13.63 ± 1.70
PLT (10^3^/uL)	193.5 ± 60.2	198.9 ± 55.4	198.9 ± 54.9	203.6 ± 61.2

BUN = blood urea nitrogen, GOT = glutamic-oxalocetic transaminase, GPT = glutamic-pyruvic transaminase, Hb = haemoglobin, PLT = platelet, WBC = white blood cell.

## 4 Discussion

In the present study, PBM + H_2_ was safe for people with PD. Considering its effects on PD symptoms, the participants experienced an improvement in the sum of UPDRS subscale scores during the intervention and a sustained benefit in mood and cognition after terminating treatment. This is the first study to investigate the safety and effectiveness of the combined use of PBM and H_2_ on PD. Although the exact molecular mechanism underlying this beneficial treatment for PD patients currently remains unknown, PBM might activate the mitochondria in neurons, facilitate adenosine triphosphate (ATP) production, and result in neuroprotective effects. The PBM treatment may also elevate the levels of ROS, which is toxic but can be selectively neutralized by H_2_ in H_2_ water. Given its small molecular size, H_2_ can pass through the blood–brain barrier, diffuse into the brain, and exert neuroprotective effects in patients with PD.

The association between mitochondrial dysfunction and PD has been well known for decades. A postmortem study demonstrated a decrease in mitochondrial subunits in the brains of people with PD.^[16]^ The impairment of mitochondria, the cellular powerhouse, leads to an energy shortage. Dopaminergic neurons in the substantia nigra are the most vulnerable to the failure of energy supply because of numerous outspreading neurites.^[17]^ Neurons are less able than muscle cells to enhance glycolysis to overcome the deficit in ATP synthesis through mitochondrial oxidative phosphorylation.^[18]^ Dysfunctional mitochondria also generate excessive ROS through the damaged electron transport chains. Free radicals attack cellular organelles and nuclei, leading to genetic mutation and cellular dysfunction. Overwhelming oxidative damage results in apoptosis, and the loss of dopaminergic neurons is inevitable.^[19]^ Several approaches may boost mitochondrial function, such as coenzyme Q10 supplement,^[20]^ aerobic exercise,^[21]^ and browning the adipose tissue.^[22]^ However, all of these are applied systemically, and their effect on the targets, dopaminergic neurons in the substantia nigra, is limited. Extracranial PBM was applied to patients with dementia, and was determined to increase ATP levels and mitochondrial function in a mouse model.^[23]^ Clinically, PBM was well tolerated by people with mild cognitive impairment and beneficial for improving the cognitive function in multiple domains and in electroencephalography and brain connectivity modalities.^[24]^ The present study used the self-designed and sophisticated projectors for generating near-infrared light to directly target the midbrain area. No participant reported a significant adverse effect from the treatment, and the skin temperature elevation was minimal, approximately 1°C to 3°C, after 30 minutes of treatment.

This study, for the first time, simultaneously administered 2 interventions (PBM + H_2_). Because ROS is an inevitable byproduct of ATP synthesis through the electron transport chain, the steady enhancement of the mitochondrial function may result in an undesirable side effect, which is excessive ROS generation. Without adequate ROS clearance, subsequent oxidative damage can trigger neuronal apoptosis. H_2_ water application is appropriate for antioxidation (Fig. 3), and H_2_ selectively reduces •OH radicals and affects numerous downstream signal transduction pathways, such as the ERK, p38, and Akt pathways.^[25]^ Moreover, H_2_ was hypothesized to be linked to the modulation of Ca^2+^ signal transduction and the nuclear factor of the activated T cell pathway.^[26]^ Although there was no direct comparison between the H_2_ water with conventional antioxidants on the antioxidative effect, H_2_ water is superior to the conventional counterparts because of the excellent cellular membrane and organelles penetration capacity and the avoidance of interfering physiological reactive species.^[27]^ With PBM + H_2_, mitochondrial ATP synthesis induction is not reduced through excessive ROS production.

The strength of this study is that we combined 2 interventions and demonstrated the safety of this regimen in people with PD. Because of the multifactorial nature of PD, the future management of PD should be more focused on a multipronged approach—similar to cocktail therapy for treating cancer or HIV infection. In addition, these 2 interventions not only target the fundamental PD pathogenesis but are also compensatory to each other: H_2_ water neutralizes an increased amount of ROS, a secondary byproduct of the boost in mitochondrial function by PBM. The present study has some limitations. This was an open-label study, and the improvement was most obvious in the mood and cognitive domains, results which were possibly biased by the placebo effect. The assessment of mood and cognition were not conducted by the conventional Beck Depression Inventory or mini-mental status examination but based on the UPDRS part I, which was not in detail like the mood and cognition specific assessment. The whole course of treatment was short because of the nature of phase I/IIa study, which focused on the safety and possible benefit of the combined therapy; hence, claiming a beneficial effect on the disease modification is difficult. Finally, H_2_ water intake mainly occurred at home and hence compliance could not be monitored.

## 5 Conclusion

The present study demonstrated that the combination regimen of PBM and H_2_ water was safe for people with PD and could alleviate disease severity, particularly in the mood and cognitive domains. In the future, a formal, longer-term, phase-II, proof-of-concept study is warranted to completely investigate the effect of the combined therapy of PBM and H_2_ water on people with PD.

## Acknowledgments

The authors appreciated Dr. Yuan-Hung Wang's help for the statistical analysis.

## Author contributions

**Conceptualization:** Chien Tai Hong, Chaur-Jong Hu, Hung-Yu Lin, Dean Wu.

**Data curation:** Dean Wu.

**Formal analysis:** Chien Tai Hong, Dean Wu.

**Funding acquisition:** Hung-Yu Lin.

**Investigation:** Chien Tai Hong, Chaur-Jong Hu, Hung-Yu Lin, Dean Wu.

**Methodology:** Chien Tai Hong, Chaur-Jong Hu, Hung-Yu Lin, Dean Wu.

**Project administration:** Dean Wu.

**Resources:** Chaur-Jong Hu, Hung-Yu Lin, Dean Wu.

**Supervision:** Chaur-Jong Hu, Hung-Yu Lin, Dean Wu.

**Validation:** Dean Wu.

**Visualization:** Hung-Yu Lin, Dean Wu.

**Writing – original draft:** Chien Tai Hong, Chaur-Jong Hu, Dean Wu.

**Writing – review and editing:** Chien Tai Hong, Chaur-Jong Hu, Hung-Yu Lin, Dean Wu.

## Abbreviations

Abbreviations: ANOVA = analysis of variance, ATP = adenosine triphosphate, H_2_ = molecular hydrogen, LED = light-emitting diode, PBM = photobiomodulation, PD = Parkinson disease, ROS = reactive oxygen species, UPDRS = Unified Parkinson Disease Rating Scale.
